# Complement, C1q, and C1q-Related Molecules Regulate Macrophage Polarization

**DOI:** 10.3389/fimmu.2014.00402

**Published:** 2014-08-21

**Authors:** Suzanne S. Bohlson, Sean D. O’Conner, Holly Jo Hulsebus, Minh-Minh Ho, Deborah A. Fraser

**Affiliations:** ^1^Department of Microbiology and Immunology, Des Moines University, Des Moines, IA, USA; ^2^Department of Biologicial Sciences, California State University Long Beach, Long Beach, CA, USA

**Keywords:** macrophage, complement, C1q, adiponectin, inflammasome, cytokine, phagocytosis, efferocytosis

## Abstract

Complement is a critical system of enzymes, regulatory proteins, and receptors that regulates both innate and adaptive immune responses. Natural mutations in complement molecules highlight their requirement in regulation of a variety of human conditions including infectious disease and autoimmunity. As sentinels of the immune system, macrophages are specialized to respond to infectious microbes, as well as normal and altered self, and dictate appropriate immune responses. Complement components such as anaphylatoxins (C3a and C5a) and opsonins [C3b, C1q, mannan binding lectin (MBL)] influence macrophage responses. While anaphylatoxins C3a and C5a trigger inflammasome activation, opsonins such as C1q and related molecules (MBL and adiponectin) downregulate inflammasome activation and inflammation, and upregulate engulfment of apoptotic cells consistent with a pro-resolving or M2 macrophage phenotype. This review summarizes our current understanding of the influence of the complement system on macrophage polarization with an emphasis on C1q and related molecules.

## Complement System

**Figure 1:**
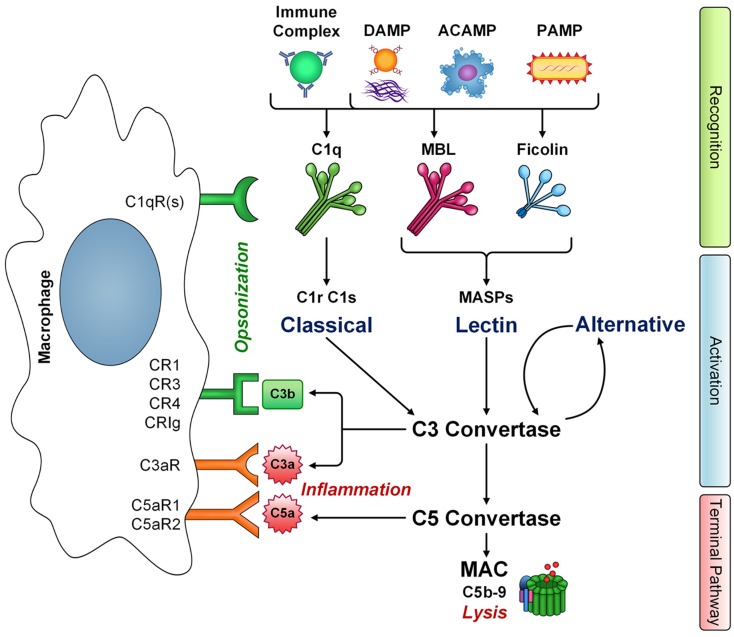
**The interaction of macrophages with complement**. Innate immune pattern recognition receptors C1q, MBL, and ficolins recognize a number of structures including immune complexes, damaged-self molecules expressing damage associated molecular patterns (DAMPS), apoptotic cells (ACAMPs), and pathogens (PAMPs), leading to activation of classical and lectin pathways, respectively, and amplification via the alternative pathway. Complement activation has three main effector functions: cytolysis via membrane attack complex (MAC) formation, inflammation mediated by anaphylatoxins C3a and C5a, and opsonization leading to phagocyte clearance via C3b deposition. However, C1q, MBL, and ficolins also have non-cascade activation related functions and are directly opsonic, leading to enhanced clearance of targets and phagocyte activation. Macrophages express a number of receptors that recognize complement components. The gC1qR and cC1qR bind the globular heads and collagen-like tail domains of C1q, respectively. However, additional C1q receptors likely exist (C1qR), some of which may also recognize the closely related collagen-like domains of other defense collagen family members, MBL and ficolins. Additional complement receptors on macrophages CR1, CR3, CR4, and CRIg recognize C3b-opsonized targets either as intact C3b or its degradation fragment iC3b. Macrophages also express receptors for complement activation fragments C3a (C3aR) and C5a (C5aR1 and C5aR2). Thus, macrophages are key players in carrying out the effector functions of complement activation.

The complement system comprises over 35 cell associated and soluble molecules, which play a critical role in our innate immune response. Activation of complement begins with a recognition step. Recognition proteins of the complement system include C1q, mannan binding lectin (MBL), and ficolins (ficolin-1, -2, -3). These proteins are innate pattern recognition receptors (PRRs) and are capable of recognizing a wide range of structures including foreign organisms, either via binding directly to their pathogen associated molecular patterns (PAMPs) or when coated with antibody in an immune-complex (C1q) [reviewed in Ref. (1)]. In addition, these PRRs also recognize and bind to structures associated with cellular damage/debris such as apoptotic cell associated molecular patterns (ACAMPs) like phosphatidylserine, damage associated molecular patterns (DAMPs) like oxidation neo-epitopes, and fibrillar protein structures (2–8). Activation of complement via the classical pathway (C1q), lectin pathway (MBL/ficolins), or alternative pathway (C3 “tickover” autoactivation/properdin) begins a coordinated cascade of enzymatic cleavage events generating complement protein fragments that carry out effector functions. These include opsonization for enhanced phagocytosis, either directly or via production of C3b, triggering inflammation through production of anaphylatoxins C3a and C5a, and lysis of target cells through deposition of C5b-9, the membrane attack complex (MAC), in the complement terminal pathway (Figure 1).

### Complement deficiencies in human disease

Genetic deficiencies in complement components highlight its important role not only in clearance of pathogens but also in removal of dying cells/cellular debris and prevention of autoimmunity. For example, genetic deficiencies in lectin, alternative and terminal pathway components like MBL, factor D, properdin, C3, C5, C6, C7, C8, and C9, increase susceptibility to infections, in particular, by encapsulated bacteria [reviewed in Ref. (9)]. The strong link between late complement component deficiencies (C5–9) and recurrent neisserial infections indicates a critical role for C5b–9 MAC deposition in direct bactericidal defense. However, deficiency in early classical pathway component C1q is strongly associated with development of the autoimmune disease systemic lupus erythematosus (SLE), likely due to impaired opsonophagocytosis, and compromised removal of immune complexes and apoptotic cells [reviewed in Ref. (10)]. Weaker associations with SLE are also seen with deficiencies in other classical pathway components C1r/s, C2, C4, and C3. Thus, complement opsonization via C1q recognition plays a critical role in maintaining normal tissue homeostasis and prevention of autoimmunity. While excessive or inappropriate complement activation is associated with almost all inflammatory or inflammation-related diseases including cancer, Alzheimer’s disease (AD), and metabolic disease [reviewed in Ref. (11, 12)], associations of these diseases with (generally very rare) complement deficiencies in humans have not been reported. However, polymorphisms in *MBL2* are common, and provide extensive evidence for links between MBL levels and cardiovascular disease [reviewed in Ref. (13)].

### Complement receptors on macrophages

Macrophages are key players in carrying out complement effector functions. Complement components including C1q, MBL, C3b, C4b, C3a, and C5a interact with a variety of receptors on macrophages, leading to modulation of cytokine production/inflammatory responses and increased opsonophagocytic clearance of targets. For example, monocytes and macrophages express complement receptors CR1 (CD35), CR3 (CD11b/CD18), and CR4 (CD11c/CD18). CR1 binds complement opsonins C1q, C3b, and C4b, which are deposited on target cells/surfaces (14) and reviewed in Ref. (15). Binding to CR1 promotes phagocytosis of targets, along with degradation of C3b to its inactive fragment iC3b, preventing C5-convertase activity and thus inhibiting the complement terminal pathway. CR3 and CR4 bind iC3b and promote the phagocytosis of targets. Genetic deficiencies in these receptors are also a risk factor for the development of SLE [reviewed in Ref. (10, 16)]. A subset of tissue macrophages expresses the complement receptor CRIg. Gene expression of this receptor is associated with activated macrophages (17), and the protein is found in human liver Kupffer cells and in subsets of various resident tissue macrophages including alveolar and synovial macrophages (18). CRIg binds to C3b, and its degradation product, iC3b, and was shown to be important in clearance of C3b-opsonized pathogens from the circulation.

Beyond CR1, macrophages express other receptors capable of binding C1q. These include the ubiquitously co-expressed molecules gC1qR and cC1qR. gC1qR binds to the globular heads of C1q (19), whereas cC1qR (calreticulin) interacts with both the collagen-like tail and globular head domains of C1q (4). C1q opsonized targets are internalized more rapidly than in the absence of C1q via interaction of the collagen-like tail with a receptor on phagocytes (20). In addition, C1q bound to a variety of targets modulates macrophage inflammatory responses via its collagen-like domain (5, 21). The collagen-like domain of C1q is a feature shared with other so-called “defense collagens.” This family of molecules includes MBL and ficolins, which also trigger enhanced phagocytosis [reviewed in Ref. (1)] and modulate cytokine production (22). While the phagocytic receptor is not definitively identified for all targets, involvement of the cC1qR has been implicated in the C1q- and MBL-mediated removal of apoptotic cells by macrophages (6). An additional family of proteins, termed C1q/TNF-related proteins (CTRPs), contains C-terminal globular domains homologous to C1q (23, 24). Adiponectin is perhaps the best characterized member of the CTRP family, and interacts with macrophages, regulating inflammatory responses similar to C1q (25). However, the receptors for the metabolic actions of adiponectin were shown to be distinct from the C1qR, and include AdipoR1 and AdiopR2 (26).

### Complement anaphylatoxins

Complement anaphylatoxins C3a and C5a are soluble complement fragments produced from C3 or C5 by activation of the C3- or C5-convertase enzyme complex, respectively. They carry out their biological functions via interactions with three receptors. These include the C3a receptor (C3aR) (27), which binds C3a but not its degradation product C3a-desArg, the C5a receptor (C5aR1) (28), which binds C5a, and C5a receptor-like 2 (C5aR2, C5L2) (29), which binds C5a but has greater affinity for C5a-desArg. C5aR2 lacks signaling capabilities (30), and thus, is considered a decoy receptor, capable of sequestering the bioavailability of C5a/C5a-desArg and limiting their ability to activate via the C5aR1. C5aR2 may also bind C3a-desArg, but this is controversial (31). Cellular expression of the anaphylatoxin receptors is widespread but particularly includes immune cells like monocytes and macrophages (32, 33). Interestingly, LPS, associated with M1 macrophage polarization increases gene expression of C5aR1 in macrophages (34) while IL-4, associated with M2 macrophage polarization downregulates C5aR1 expression (35).

## Macrophage Polarization

Macrophages are grouped as M1 and M2 in accordance with the Th1/Th2 nomenclature, and this terminology describes two macrophage phenotypes: the pro-inflammatory/classically activated macrophage (M1) and the pro-resolving/alternatively activated macrophage (M2). The expression of a variety of genes has been associated with macrophage polarization, most notably, the machinery required for enzymatic breakdown of arginine in pro-resolving and pro-inflammatory macrophages. By expressing arginase, pro-resolving macrophages generate ornithine, which promotes proliferation and repair, whereas pro-inflammatory (M1) macrophages express inducible nitric oxide synthase and generate nitric oxide (NO), an important molecule in host defense against invading pathogens, which also inhibits cell proliferation [reviewed in Ref. (36)]. Intermediates in the two enzymatic pathways act antagonistically, inhibiting the other when they are activated (37). Consistent with a role in promotion of inflammation, M1 macrophages are also often associated with an increased production of pro-inflammatory cytokines such as TNFα and IL-1β. In contrast, M2 macrophages, which are pro-resolving, are often associated with increased production of anti-inflammatory cytokine IL-10.

Although the idea of macrophages having separate phenotypes is helpful in defining their action, it is also somewhat misleading and over simplified. *In vivo*, macrophages are constantly encountering various external signals leading to a very fluid existence in terms of phenotypes (38). Even within the same site and population, macrophages can express different and constantly changing phenotypes, termed macrophage plasticity (39). Macrophage polarization is a result of a combination of external signals macrophages receive from their environment. Common signals that have been investigated include the Th1 cytokine interferon-γ (IFN-γ) and various PAMPs, which are associated with M1 polarization and the Th2 cytokines IL-4/IL-13, which are associated with M2 polarization. Here, we will review some recent work illuminating the role of the complement system in regulating macrophage activation and polarization.

## Complement Anaphylatoxins Regulate Macrophage Activation

Complement anaphylatoxins, C3a and C5a, are pro-inflammatory and trigger monocyte and macrophage activation through various signaling mechanisms. For example, upon LPS stimulation in human monocytes, C3a induces NLRP3 inflammasome activation through an increase in ATP release mediated by extracellular signal-regulated kinase 1/2 (ERK1/2) (40). Samstad et al. found that C5a produced during complement activation by cholesterol crystals, influenced inflammation through NLRP3 inflammasome activation and IL-1β and TNFα release, and increased the production of reactive oxygen species (ROS) (41). C5a was also correlated with IL-6 induction and development of inflammatory T-helper 17 cells (42), as well as affecting IL-17 and IL-23 production (43). Interestingly, sublethal MAC (C5b-9) deposition rather than C3a or C5a was demonstrated to trigger inflammasome activation in murine dendritic cells following LPS stimulation (44). In the CNS, C5a provides a chemotactic and activation signal for microglia and astrocytes [reviewed in Ref. (45)]. It can also synergize with damage signals such as amyloid beta (Aβ) to trigger enhanced inflammatory cytokine production (46). Thus, activation of complement by extracellular Aβ plaques can exacerbate inflammation and may play a substantial role in the pathogenesis of AD. Indeed, treatment with a C5a receptor antagonist was shown to decrease deposition of fibrillar Aβ and inflammatory glia and improve cognitive performance in mice models of AD (47). Many other diseases are associated with anaphylatoxin signaling, including allergic, infectious, autoimmune diseases, and cancer (48). Clearly, the pro-inflammatory signaling provided by the anaphylatoxins contributes to both beneficial (pathogen clearing) and detrimental (inflammatory disease-related) inflammation.

## Complement Opsonins Regulate Macrophage Activation

### C3b-mediated opsonization

One of the major effector functions of the complement system is the tagging, or opsonization, of pathogens and/or cellular debris for clearance by phagocytes. Complement component C3 is the most abundant complement component in blood at about 1.2 mg/ml, and as such, permissible surfaces become readily coated in C3b and iC3b following cleavage of C3. CR3 is a major phagocytic receptor expressed on macrophages that is involved in clearance of iC3b opsonized particles (Figure 1). In contrast to other phagocytic receptors such as Fcγ receptors, engulfment of iC3b coated particles via CR3 has traditionally been considered anti-inflammatory. For example, CR3-dependent engulfment does not activate the arachidonic acid cascade (49) or the release of toxic oxygen products (50). In early experiments assessing macrophage heterogeneity, Stein and colleagues demonstrated increased secretion of TNFα from macrophages elicited into the peritoneal cavity by inflammatory mediators (e.g., thioglycollate) when compared to resident peritoneal macrophages. However, independent of the macrophage phenotype, all macrophages failed to secrete significant levels of TNFα following ligation of CR3 whereas ligation of Fcγ receptors led to TNFα release by all macrophage subsets (51). In studies with the macrophage intracellular pathogen *Mycobacterium avium*, C3-independent phagocytosis of *M. avium* resulted in enhanced TNFα production (52, 53). More recent reports assessing pathogenesis of *Francisella tularensis* support a role for CR3 in inducing immune suppression and facilitating infection with this intracellular pathogen (54). While CR3-dependent immune suppression is detrimental in the course of *F. tularensis* infection or other infectious disease processes, it is beneficial in the context of clearance of apoptotic cells and/or cellular debris.

### Complement-dependent engulfment of apoptotic cells

**Figure 2:**
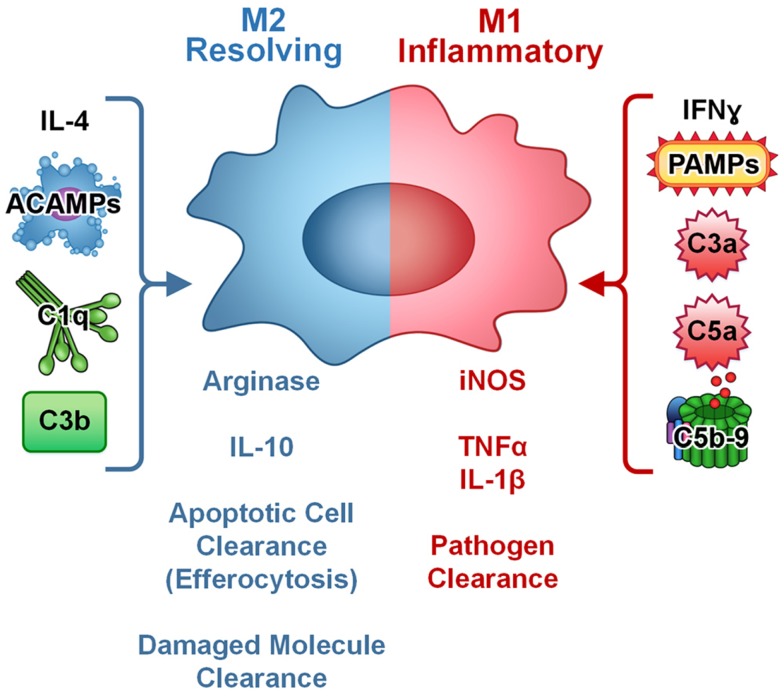
**Complement regulates macrophage polarization**. In culture, M1 macrophages (inflammatory, pathogen clearing) are induced by IFNγ or PAMPs and are characterized by production of iNOS and pro-inflammatory cytokines such as TNFα and IL-1β. M2 macrophages (resolving, apoptotic cell and damaged molecule clearing) are induced by IL-4 and characterized by arginase and anti-inflammatory cytokine IL-10 production. Complement components C3a, C5a, and C5b-9 modulate cytokine production in macrophages toward an inflammatory (M1-like) phenotype. Apoptotic cells and targets opsonized with complement components C1q or C3b increase clearance and modulate cytokine production in macrophages toward an anti-inflammatory, resolving (M2-like) phenotype and can block PAMP-mediated pro-inflammatory signaling. Thus, complement plays a dual role in macrophage activation and polarization depending on the target.

Complement components readily coat the surface of apoptotic cells and facilitate ingestion by macrophages (55, 56). Ingestion of apoptotic cells is a silent process accompanied by the production of anti-inflammatory cytokines TGFβ and IL-10 (57). Complement mediated opsonization of apoptotic cells is largely dependent on the classical complement pathway, and deficiencies in early components of the classical pathway (C1q, C4, and C2) result in inefficient disposal of apoptotic cells and subsequent autoimmunity (58–60). While C1q deficiency results in development of lupus in virtually all cases, absence of C3, C2, and C4 results in lupus at lower frequency indicating a role for C1q beyond classical complement pathway activation in regulation of the immune response (61). Recent reports have indicated that C1q regulates the monocyte/macrophage/dendritic cell phenotype leading to development of a phagocyte that is specialized to resolve inflammation. Specifically, the C1q-stimulated phagocyte is pro-efferocytic and anti-inflammatory (Figure 2). As such, complement opsonins are mediating activity beyond the immediate stimulation of enhanced phagocytosis; they are inducing a macrophage phenotype, or polarizing macrophages toward a pro-resolving phenotype. This would be consistent with observation that C1q deficiency results in autoimmunity and chronic inflammation.

Korb and Ahearn were the first to describe a role for C1q in the clearance of apoptotic cells, and suggested that C1q-dependent engulfment of apoptotic cells was important in prevention of autoimmunity in lupus (62). There has been wide support for this hypothesis, and Bhatia et al. demonstrated that removal of apoptotic cells by C1q was also important in prevention of the inflammatory disease atherosclerosis (63). However, the receptors/signal transduction pathway leading to C1q-dependent efferocytosis has not been clearly delineated [reviewed in Ref. (56)]. C1q binds to apoptotic cells and serves as a bridging molecule linking the apoptotic cell to the phagocyte via calreticulin (cC1qR) and its binding partner, the phagocytic receptor LRP (CD91) (6). However, macrophages deficient in LRP still respond to C1q with enhanced phagocytosis and efferocytosis indicating that there are multiple mechanisms of C1q-dependent engulfment (64). We demonstrated that mouse bone marrow derived macrophages and peritoneal macrophages stimulated with C1q upregulated expression of engulfment machinery including Mer tyrosine kinase and the MerTK ligand Gas6, leading to development of a macrophage that is primed for efferocytosis (65). More recently, we showed that this pathway is shared with a C1q homolog, adiponectin (66) and not with MBL, a C1q-related collectin (65).

### C1q and adiponectin mediate efferocytosis via a shared pathway

Adiponectin is referred to as an adipokine; it is produced by adipocytes and is secreted into circulation where it modulates biological responses via several receptors including adiponectin receptor 1 (AdipoR1), adiponectin receptor 2 (AdipoR2), or T cadherin (T-cad) (67, 68). More recent studies have suggested there is an additional receptor on macrophages that mediates adiponectin signaling; however, this receptor has not been identified (69). Adiponectin signaling leads to activation of 5′ adenosine monophosphate-activated protein kinase (AMPK) and peroxisome proliferator-activated receptor alpha (PPARα), and adiponectin-dependent regulation of metabolism is mediated via these key signaling nodes (70). Much information has been gathered regarding the role of AMPK in the regulation of metabolic activity; however, recent studies suggest that AMPK also influences the immune response, including macrophage cytokine expression and phagocytosis. For example, silencing of AMPK inhibits LPS- and fatty acid-mediated inflammation in macrophages (71). In addition, AMPK activation is associated with enhanced phagocytosis and efferocytosis, as well as macrophage polarization (72, 73). Similarly, C1q stimulates enhanced phagocytosis and diminution of pro-inflammatory cytokine production from myeloid cells, and we recently demonstrated that C1q and adiponectin-dependent Mer expression and efferocytosis require activation of AMPK (66). These studies have begun to define the mechanism by which C1q and related opsonins modulate macrophage activation.

### C1q and MBL inhibit pro-inflammatory and promote anti-inflammatory cytokine production

In line with these observations, Fraser and colleagues demonstrated that both C1q-stimulated human monocytes and C1q-stimulated mouse microglia produce increased anti-inflammatory IL-10 and decreased pro-inflammatory cytokines following stimulation with the TLR4 ligand LPS (21, 22). Similar activity was shown for MBL indicating that this is a distinct mechanism for macrophage activation, independent of AMPK-mediated Mer expression since MBL failed to upregulate Mer-dependent efferocytosis. C1q/MBL-dependent activation of NFκB p50p50 homodimers were suggested to contribute to the anti-inflammatory phenotype via competitive inhibition of pro-inflammatory NFκB p50p65 heterodimer activation and/or via transcriptional activation of IL-10 (74). The same group demonstrated that C1q promoted M2 polarization and limited inflammasome activation in human monocyte derived macrophages (75). Interferon-alpha (IFN-α) is a pro-inflammatory cytokine that contributes to dendritic cell activation, a breakdown in peripheral tolerance and autoimmunity (76). C1q modulates IFN-α production from human phagocytes in response to a variety of stimuli (75, 77, 78). Santer and colleagues demonstrated that C1q deficiency in human lupus patients resulted in elevated IFN-α levels in serum and cerebrospinal fluid, and that C1q suppressed immune-complex stimulated IFN-α production from human monocytes (78–80). The anti-inflammatory effects of C1q are not limited to apoptotic cells. C1q has also been shown to enhance the uptake of atherogenic forms of lipoproteins such as oxidized or acetylated LDL (oxLDL, AcLDL) (81). During clearance of oxLDL by macrophages, C1q also modulates cytokine production toward an anti-inflammatory, resolving phenotype and dampens transcriptional activity by p50/p65 NFκB heterodimers, which may be important in limiting inflammation in the early atherosclerotic lesion (82). Combined, these data further support the hypothesis that C1q programs macrophages toward an anti-inflammatory, pro-efferocytic/phagocytic phenotype. Future studies should delineate the relative activity of C1q-dependent constitutive efferocytosis/phagocytosis versus programed polarization in the contribution toward protection from autoimmune and inflammatory disease.

## Concluding Remarks

The complement system has traditionally been considered an arm of the innate immune response required for promotion of inflammation and pathogen clearance. While these functions of complement are essential to host defense, more recent advances demonstrate a novel role for components of the complement system in resolution of inflammation and protection from autoimmune and inflammatory diseases including SLE, neurodegenerative disease, and atherosclerosis. In particular, complement component C1q directs macrophage polarization leading to generation of pro-resolving macrophages that promote clearance of apoptotic cells with diminished pro-inflammatory cytokine production and increased anti-inflammatory cytokine production. C1q is synthesized by macrophages in response to tissue injury and is likely to be an important signal in resolution of inflammation independent of other complement components. Moreover, C1q-related molecules such as the complement component MBL, and the adipokine adiponectin, also downregulate macrophage-mediated inflammatory responses and upregulate efferocytosis. Identification of the molecular mechanisms by which these molecules govern macrophage activation, as well as their relative contribution to disease resolution, should reveal pathways to target for development of novel therapeutics in autoimmune and inflammatory disease.

## Author Contributions

Suzanne Slater Bohlson outlined the review, invited co-authors, wrote the section on complement opsonins, extensively edited all sections, and prepared the manuscript for publication. Sean David O’Conner drafted the section on macrophage polarization. Holly Jo Hulsebus drafted the section on complement anaphylatoxins. Minh-Minh Ho designed and created the figures and figure legends. Deborah Ann Fraser wrote the section on complement and extensively edited the entire manuscript. All authors reviewed the final document prior to submission.

## Conflict of Interest Statement

The authors declare that the research was conducted in the absence of any commercial or financial relationships that could be construed as a potential conflict of interest.
